# Implications of Tissue Engineering for Tendon Repair and Regeneration

**DOI:** 10.3390/jfb16110403

**Published:** 2025-10-28

**Authors:** Dana Ivanisova, Katarina Bevizova, Sara Vach Agocsova, Lubos Danisovic, Martina Culenova

**Affiliations:** 1 Institute of Medical Biology, Genetics and Clinical Genetics, Faculty of Medicine, Comenius University, Sasinkova 4, 811 08 Bratislava, Slovakia; dana.ivanisova@fmed.uniba.sk (D.I.); lubos.danisovic@fmed.uniba.sk (L.D.); 2 Institute of Anatomy, Faculty of Medicine, Comenius University, Sasinkova 2, 811 08 Bratislava, Slovakia; katarina.bevizova@fmed.uniba.sk; 3 Institute of Natural and Synthetic Polymers, Faculty of Chemical and Food Technology, Slovak University of Technology, 812 37 Bratislava, Slovakia; sara.agocsova@stuba.sk; 4 National Institute of Rheumatic Diseases, Nabrezie I. Krasku 4, 921 12 Piestany, Slovakia; 5 Regenmed Ltd., Medená 29, 811 02 Bratislava, Slovakia

**Keywords:** tissue engineering, tendons, cells, scaffolding material, additive manufacturing

## Abstract

Tendon injuries affect millions of people globally and are among the most prevalent musculoskeletal conditions, frequently resulting in chronic pain, reduced mobility, and functional impairment. While conservative and surgical treatments are available, limitations such as low healing capacity, scar formation, and reduced biomechanics necessitate alternative approaches. Tissue engineering offers a promising solution by combining cells, scaffolds, and bioactive molecules to regenerate tendon tissue. This review presents key concepts and emerging trends, highlighting the cellular components, scaffold materials, and manufacturing processes. Tenocytes and mesenchymal stem cells are fundamental for tissue regeneration, as they synthesize extracellular matrix components and regulate inflammatory responses. Various natural and synthetic polymers have been fabricated into scaffolds that mimic the structure and biomechanics of natural tendons. Composite and hybrid scaffolds are utilized to improve the biocompatibility of natural materials with the mechanical stability of synthetic materials. Advanced technologies, such as electrospinning, freeze-drying, and 3D bioprinting, enable the creation of scaffolds with defined architecture and functional gradients, improving cell alignment, differentiation, and tendon–bone integration. Although promising preclinical data exists, major challenges remain in translating these strategies clinically, particularly vascularization, immune rejection, and mechanical stability. Continued interdisciplinary attempts in biomaterials science, cellular biology, and engineering are crucial to advancing clinically viable tendon tissue engineering.

## 1. Introduction

Tendons are fibrous, highly organized, load-bearing connective tissues that anchor muscles to bones. They mainly consist of water (55–70%), aligned type I collagen fibers, and various proteins including elastin, decorin, biglycan, and fibromodulin. Their primary function is to enable locomotion by transmitting contractile force from muscles to bones. Tendon injuries are a common musculoskeletal disease affecting millions of people annually. While not life-threatening, these injuries cause significant pain, swelling, and disability, diminishing the quality of life and generating a substantial financial burden to healthcare systems [1].

Tendon disorders result from an interplay of intrinsic and extrinsic factors. Aging is a significant factor associated with the breakdown of collagen. Damaged collagen fragments accumulate in the tendon, affecting its mechanical properties and thereby increasing the risk of injury. Sex hormones also impact tendon health, with a balance of estrogen and testosterone being crucial for tendon integrity. Endocrine imbalances can increase the risk of injury, with men being more susceptible. Obesity is another predisposing factor that contributes to tendon problems by producing harmful peptides, including chemerin, leptin, and adiponectin, from adipocytes. Diabetes type 2 also increases the risk of tendinopathy and rupture despite reduced physical activity. Elevated blood glucose promotes the formation of advanced glycation end-products in the tendon, which can cause it to stiffen and weaken. Some medications can also damage tendons. For instance, fluoroquinolone antibiotics increase the risk of tendon rupture, particularly in older patients or when applied in combination with corticosteroids. While corticosteroids are commonly used to treat tendon disorders, they can also cause tendon ruptures [2].

Spontaneous healing of minor injuries without surgery; however, the process is slow, and the formed scar tissue is weak and remodels itself over time [1]. Conservative approaches, including rest, exercise, cold therapy, NSAIDs, corticosteroids, ultrasound, and laser therapy, can be beneficial. However, reduced blood supply and low cell density restrain the spontaneous healing of tendons. Serious injuries, such as a complete rupture, usually require surgery, with associated risks of infection, scarring, necrosis, and nerve damage [3]. Despite the frequency and severity of tendon injuries, effective therapies to restore physiological function remain limited. Although all tendons heal through fibrotic processes, intrasynovial tendons present a particular challenge, as adhesions often develop and restrict mobility [4].

**Figure 1:**
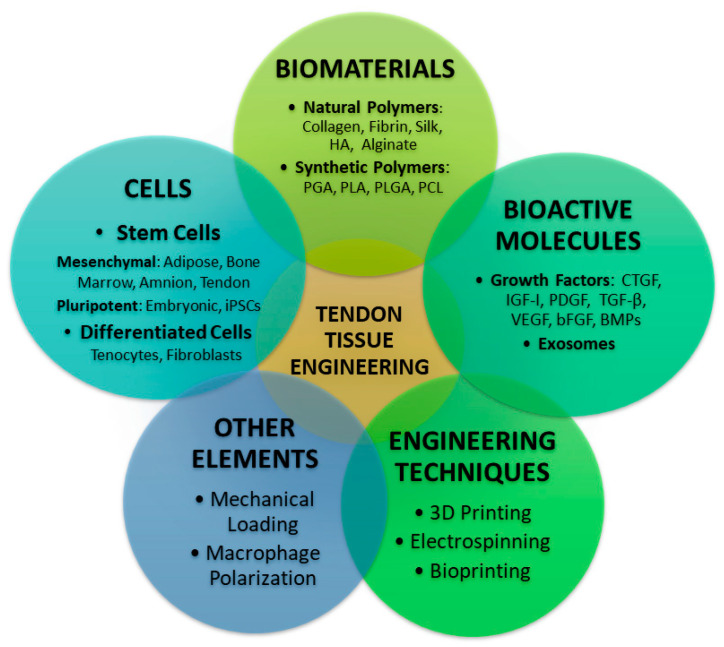
The main pillars of tendon TE represent biomaterials, cells, bioactive molecules, engineering techniques, and other elements such as mechanical stimulation and immune modulation. The interplay of these factors is essential for development of functional and mechanically competent tendon structure. Figure was drawn by authors in Microsoft Powerpoint.

To address these challenges, tissue engineering (TE) has emerged as a favorable approach (Figure 1).

It involves the use of cells, scaffolds, and bioactive molecules to replace damaged tendon tissue and stimulate the growth of new, functional tissue. The main principles of these techniques involve designing a scaffold that closely resembles the original tendon’s physical and mechanical properties, using a mix of cells (tenocytes, fibroblasts, and other specialized cells) to mimic the tendon’s composition, and creating an environment that supports cell survival as well as extracellular matrix (ECM) synthesis [5].

The main aim of the present paper is to deliver a narrative review of the most recent advancements in tendon TE, with emphasis on the biological principles, cellular components, scaffold materials, and fabrication techniques involved in tendon repair and regeneration. A comprehensive review was conducted on PubMed to identify studies focused on all aspects of tendon TE using the following search terms: “tissue engineering,” “tendons,” “cells,” “tenocytes,” “stem cells,” “scaffolding material,” “polymers,” “additive manufacturing,” “3D bioprinting”; “healing”. All these search terms were queried using combinations of the Boolean operators “AND” or “OR.” The search was limited to the last 25 years, the English language, and studies performed on humans or animals.

## 2. Anatomic and Histologic Considerations

Tendons form functional anatomical structures that participate in the movement. They are positioned between muscles and bones and attach the muscle to the bone at two points—proximal attachment, so-called origin, and the distal attachment, so-called insertion. Muscle contractions are transmitted to the bone through these connections. A healthy tendon adapts to the morphological properties of the muscle, thereby allowing its proper functioning and preventing muscle injury. The shape, size, and strength of tendons depend on the location and the function of the muscle. Longer and tighter are the tendons of the muscles belonging to finer movements (tendons in the hand and foot), and shorter and broader are the tendons of muscles generating a lot of force (e.g., Achilles tendon). Flattened tendons of any type are referred to as aponeuroses, such as those found in the abdominal muscles [6].

Tendons are highly specialized connective tissues with a crucial role in transmitting force between muscles and bones. Their composition is primarily made up of collagen fibers, with collagen type I being the most prevalent (approximately 65–80% of the tendon’s dry weight). This structural protein forms long, parallel fibers that contribute to the tensile strength and durability of tendons, allowing them to resist significant loads during movement. Tendons also contain specialized fibroblast-like cells, known as tenoblasts and tenocytes [7]. Tenoblasts are immature, proliferative, fibroblast-like cells responsible for producing collagen and producing the ECM. They are particularly abundant in developing tendons and play a fundamental role in growth and remodeling. They differentiate into tenocytes, which are elongated, spindle-shaped cells embedded within the collagen fibers. Tenocytes maintain tendon homeostasis by regulating ECM turnover and responding to mechanical stress and biochemical signals [8].

Tendons also contain other ECM elements, including elastin, proteoglycans, glycoproteins, and inorganic components. Elastin is present in small amounts (1–2% of tendon dry weight), plays a significant role in tendon flexibility and recoil, thereby preventing excessive stiffness. Proteoglycans, such as decorin, biglycan, and fibromodulin, regulate the organization and hydration of collagen fibers, thereby influencing the viscoelastic characteristics of tendons. Glycoproteins, including tenascin-C, are involved in cell adhesion, migration, and maintaining ECM integrity. In addition, tendons contain small amounts of inorganic components, including calcium, magnesium, and phosphate, which contribute to tissue stability and biochemical signaling [7].

**Figure 2:**
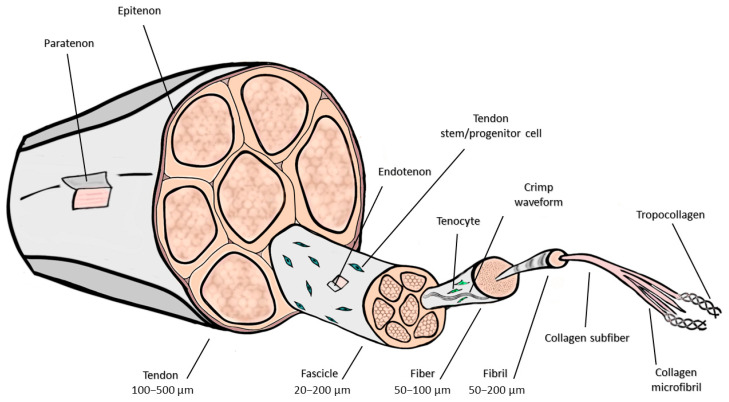
Hierarchical structure of the tendon, showing its multi-level organization. The endotenon separates individual fascicles, each containing bundles of aligned collagen fibers. Each fiber is composed of smaller fibrils, which in turn are formed by collagen subfibrils. Within the subfibrils, collagen microfibrils are organized from tropcollagen. Figure was drawn by authors in Microsoft Powerpoint and GIMP.

The histological structure of tendons follows a hierarchical organization that enables them to withstand mechanical loading and tensile forces (Figure 2). At the most minor level, collagen fibrils, formed of triple-helical type I collagen, form collagen fibers that provide load-bearing capacity. These fibers exhibit a 67 nm periodic banding pattern and follow a wave-like crimp structure, allowing tendons to stretch and absorb forces. Groups of fibers form fascicles, which are ensheathed by endotenon, a connective tissue layer containing blood vessels, lymphatics, and nerves. The tendon proper consists of multiple fascicles encased within the epitenon, while the paratenon, a loose connective tissue layer, facilitates gliding and reduces friction against surrounding structures [7].

Tendons undergo continuous ECM remodeling under mechanical stress, injury, and aging. The balance between collagen synthesis and degradation, governed by the matrix metalloproteinases (MMPs) and tissue inhibitors of metalloproteinases axis, is crucial for maintaining tendon integrity. During adaptation and repair, MMPs degrade damaged collagen, while fibroblasts and tenocytes synthesize new fibers. However, due to their limited vascularization, tendons have a reduced capacity for regeneration, making them liable to degeneration and chronic injury [9].

Anatomically, a tendon has three coverings: epitenon, endotenon, and paratenon. Longitudinally arranged collagen fibers form a dense connective tissue. The fibers run parallel to each other and are joined into fascicles, which together form a tendon. Fascicles are ensheathed by connective tissue, which holds them together and brings blood vessels and nerves to the tendon (endotenon). The tendon is covered at its surface by a thin connective-tissue sheath, the epitenon. The epitenon, which receives its lymphatic, blood, and nerve supply, extends into the tendon between collagen fascicles and the endotenon. The epitenon is covered by the paratenon, a layer of connective tissue that allows the tendon to move against the epitenon and the surrounding structures. Together, the paratenon and epitenon are collectively referred to as the peritenon [10].

Certain tendons, particularly those that experience frequent movement and high friction, are enclosed within tendon sheaths—double-layered tubular structures formed of an outer fibrous layer and an inner synovial membrane. The synovial fluid within the sheath minimizes friction, ensuring smooth tendon movement. These sheaths occur in tendons that pass through narrow spaces, such as those in the wrist and fingers. Other anatomical structures surrounding tendons include bursae, joint capsules, pulleys, fat pads, and retinacula, which collectively help reduce pressure and friction, ensuring optimal tendon function [10].

The vascular supply of the tendons is significantly lower than the supply of the muscles, which determines the whitish color of the tendons. During development, the tendons are supplied by an extensive capillary network; however, the vascular supply of the mature tendon is significantly lower. Tendons also contain so-called avascular zones, which have minimal or no blood supply. These zones are particularly prone to reduced regeneration and an increased risk of injury, as insufficient vascularization limits the delivery of oxygen and nutrients necessary for healing [11].

Branches of muscular, cutaneous, and peritendinous nerves innervate tendons. These nerves are located within the surrounding structures, including the paratenon, epitenon, and endotenon, whereas the tendon proper is almost devoid of direct neuronal supply. The terminal branches of these nerves are classified as myelinated or unmyelinated based on their anatomical structure. Myelinated fibers are primarily located near the muscle and function as mechanoreceptors, detecting tension and pressure in the tendon. In contrast, unmyelinated fibers serve as nociceptors responsible for pain perception and transmission [10].

## 3. Cells Involved in Tendon Tissue Engineering

**Figure 3:**
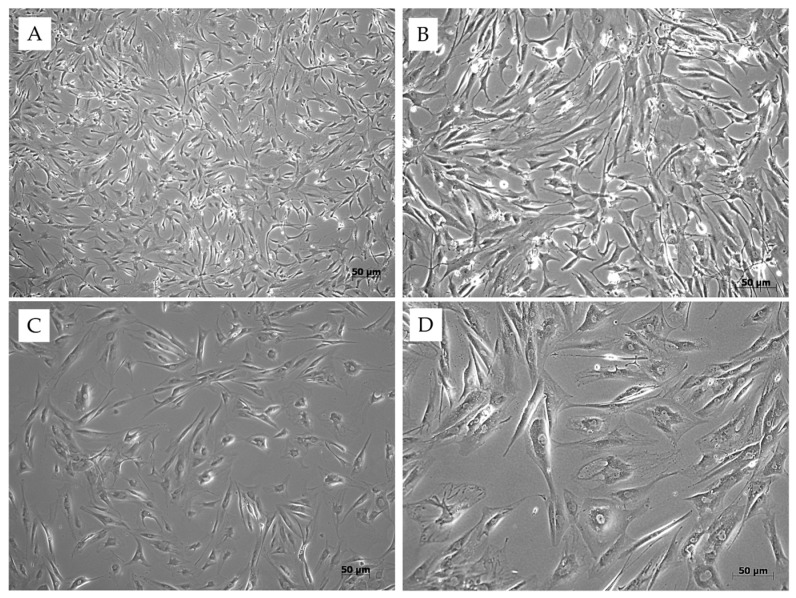
Morphology of tenocytes and adipose-derived mesenchymal stem cells (AD-MSCs). (**A**,**B**) Tenocytes at passage 1 exhibit an elongated, spindle-shaped morphology typical of tendon fibroblasts, showing formation of interconnected cellular networks. Images were taken at 50× (**A**) and 100× (**B**) magnification. (**C**,**D**) Adipose-derived mesenchymal stem cells (AD-MSCs) at passage 3 display a fibroblast-like morphology with elongated cytoplasmic processes and a uniform, spindle-shaped appearance characteristic of mesenchymal stem cells. Images were captured at 50× (**C**) and 100× (**D**) magnification using a Zeiss Axiovert 100 inverted microscope (Carl Zeiss, Jena, Germany) equipped with a digital camera. All images were captured by Dana Ivanisova.

Tenocytes are highly differentiated cells that can proliferate both in vivo and in vitro (Figure 3). They represent the most abundant cell population within healthy tendons, making them an essential component for tendon regeneration. They are naturally located between collagen fibers and the interfascicular matrix, participating in tendon repair by producing growth factors and ECM [12,13].

Sevivas et al. [14] found that a substance secreted by human bone marrow stem cells (BMSCs) can improve the survival and growth of human tendon cells in vitro. When these enhanced tendon cells were placed on a specific type of scaffold and used to regenerate a significant rotator cuff injury in rats, the researchers observed an increase in cell density and the formation of fibrocartilage-like tissue at the junction of the tendon and bone. The repaired tendon was also stronger and more flexible. Lohan et al. [15] reseeded decellularized porcine Achilles tendons with human tenocytes and implanted them into mice. They remained unchanged in size after 6–12 weeks, showing no signs of inflammation or encapsulation. Cell distribution was more uniform in vivo compared to in vitro conditions. When reseeded with tenocytes, the ECM scaffold achieved higher histological scores than the cell-free ECM. Human tenocytes could be identified within the explants. Experimental results also demonstrated the formation of neo-tendon tissue.

However, harvesting tenocytes from healthy tendons can damage normal tendon tissue. The following limitation is the low cell density of tendon tissue, which makes it challenging to obtain enough tenocytes. Moreover, tenocytes often undergo dedifferentiation during in vitro expansion, changing their shape from a spindle-like to a more rounded tenoblast-like appearance. Dedifferentiation suppresses collagen biosynthesis and the formation of other ECM constituents [16].

Owing to the challenges associated with tenocytes, tendon TE predominantly employs adult tissue–derived mesenchymal stem cells (MSCs). MSCs are adult, multipotent, non-hematopoietic progenitor cells that can adhere to plastic surfaces, self-renew, and exhibit a spindle-shaped morphology [16]. They are characterized by the expression of specific antigens CD73, CD90, Sca-1, and CD105. They should not express CD11b, CD14, CD19, CD34, CD45, or HLA-DR. MSCs can be isolated from various tissues, including adipose tissue, bone marrow, umbilical cord, dental pulp, skeletal muscle, synovial fluid, and many others. While MSCs can differentiate into different cell types of mesodermal origin, evidence suggests their ability to activate genes associated with specialized ectodermal and endodermal cells [16,17].

MSCs primarily mediate tissue repair through paracrine actions rather than solely differentiating into new cells. They release various factors into their environment, including growth factors, cytokines, chemokines, ECM components, antioxidants, and extracellular vesicles (EVs). This ‘MSC secretome’ creates a favorable environment for stimulating tissue regeneration and modulating the immune response [18]. MSCs can reduce inflammation by influencing macrophage polarization and promoting the recruitment of anti-inflammatory M2 macrophages [19]. Studies show that EVs from AD-MSCs and human amniotic membrane–derived MSCs (AM-MSCs) play an important therapeutic role in chronic rotator cuff tendinopathy. These EVs can reduce inflammation by shifting macrophages from a pro-inflammatory M1 phenotype to an anti-inflammatory M2 phenotype [20]. The secretome of human AM-MSCs has shown promising results as a cell-free therapy for treating musculoskeletal conditions, including tendinopathy. Ragni et al. [21] identified 37 cytokines/chemokines in the human AM-MSC secretome; several mediate inflammatory-cell chemotaxis and homeostasis and contribute to ECM remodeling. EV-miRNA analysis further indicated suppression of inflammatory T cells and promotion of regulatory T cells.

McClellan et al. [22] showed that MSCs seeded on an electrochemically aligned collagen scaffold improved biomechanical and histological outcomes in critical-size rotator cuff defects in a rabbit model.

## 4. Scaffolds Used for Tissue Engineering of the Tendon

**Figure 4:**
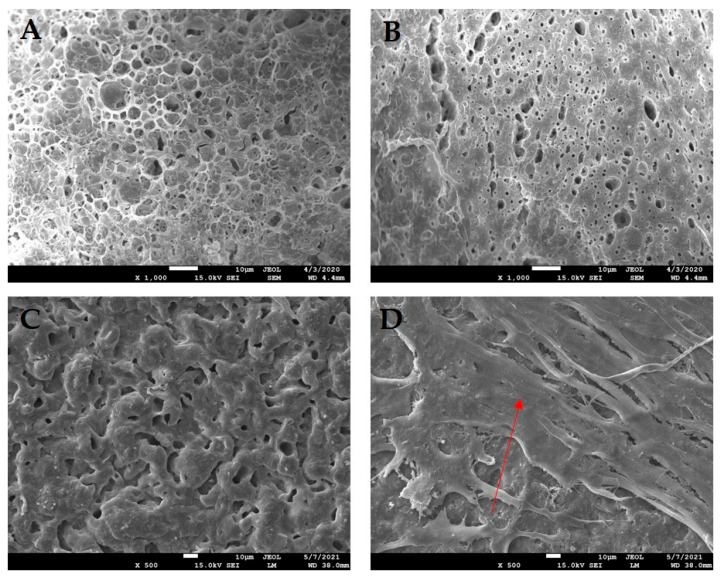
Scaffolds. Scanning electron microscopy (SEM) of various scaffolds applied in TE. (**A**): The cross-section of starch-based planar scaffold; (**B**): cross-section of PLA-based planar scaffold; (**C**): surface of a PLA scaffold before cell seeding; (**D**): surface of a PLA scaffold after seeding with adipose tissue–derived MSCs. The red arrow indicates that cells could attach to the surface and proliferate, forming a dense cellular layer. All SEM micrographs were captured using a JEOL 7500F scanning electron microscope (JEOL Ltd., Tokyo, Japan) operated by Martina Culenova.

TE combines cells with scaffolds to mimic the native ECM’s mechanical and chemical properties. Bioactive molecules then stimulate cell growth [23]. A comprehensive understanding of biomimetic materials is critical for developing scaffolds that facilitate the desired cellular responses. In tendon TE, scaffolds must be multifunctional to enhance mechanical properties, improve cell signaling, and promote cell adhesion. Various combinations of biomaterials are employed to mimic the natural characteristics of tissue in vitro (Figure 4). Using appropriate fabrication techniques and cell loading, surrogate structures for musculoskeletal system repair can be prepared [24].

Scaffolds must generally fulfill fundamental properties such as biocompatibility, biodegradability, porous structure, and mechanical support [25]. Additionally, more specific features are derived from their intended application. TE for tendon regeneration primarily involves the production of scaffolds with physicochemical and biomechanical attributes that closely resemble those of native tissue. Selecting appropriate cell types (tenocytes, MSCs, fibroblasts) is critical to mimic tendon cellularity and support tenocyte survival and ECM synthesis within the scaffold [5]. Specifically, oriented fibers and fiber bundles characterize the ultrastructure of the tendon. Novel computer-aided technologies, such as 3D printing, enable scaffolds to mimic the tissue’s physiological ultrastructure, providing a suitable environment for optimal cellular behavior, ECM production, and integration with adjacent tissues [26]. Furthermore, scaffolds can be loaded with various bioactive molecules to enhance and control these processes [27].

### 4.1. Natural Scaffolds

Scaffolds belonging to this group are derived from decellularized native tendons or native polymers. These materials offer advantages such as low immunogenicity, biocompatibility, and biodegradability [28]. Due to their biological origin, they can closely resemble the native tissue structure, promoting favorable cell-scaffold interactions. However, their harvesting often requires invasive procedures on living organisms [25].

#### 4.1.1. Collagen

Collagen is the predominant ECM component in human tendons and ligaments and was among the first natural polymers used for tendon-regeneration scaffolds [29]. Collagen-based scaffolds provide abundant binding sites for cells and growth factors, promoting adhesion, proliferation, migration, and differentiation [30]. Collagen matrices featuring highly aligned fibers show promise for tendon TE owing to their biomimetic composition and anisotropic architecture. Moreover, insoluble collagen fibers are more suitable for scaffolding than soluble ones as they self-assemble to achieve mechanical properties comparable to the native ECM [31]. Collagen utilized for scaffold fabrication is predominantly derived from animal tissues. To ensure immunotolerance and improve mechanical properties, it is essential to eliminate all antigens and pathogens through chemical crosslinking [32]. When processed, this natural polymer exhibits favorable characteristics, including a porous structure, low immunogenicity, good permeability, biocompatibility, and biodegradability. Nonetheless, collagen-based scaffolds can lack mechanical strength and stability in aqueous media. These shortcomings can be mitigated by crosslinking and by blending with other natural or synthetic polymers [33].

Fiber alignment within the collagen scaffold’s structure plays an important role, as it can significantly enhance its biological and mechanical characteristics. Yang et al. [34] applied rotating extrusion technology to produce collagen scaffolds with aligned or unaligned fibers from insoluble collagen. The aim was to determine how fiber orientation influences the differentiation of seeded BMSCs in vitro and in vivo tendon repair. Better tenogenic differentiation (higher expression of tendon-specific genes and changes in cell morphology) and in vivo repair of rat Achilles tendons were observed when fibers were oriented and narrowly distributed (orientation angle 0–15°).

To obtain aligned collagen fibers, techniques such as electrospinning, electrochemical methods, and microfluidic methods have been utilized [35]. These approaches employ reconstituted (denatured) collagen, which diminishes biomechanical strength and hastens in vivo breakdown. Additionally, these approaches face difficulty in achieving complete decellularization, which leads to residual immunogenicity and pathogen risk, thereby reducing clinical favorability [36].

A Roßbach et al. [37] study combined autologous tenocytes with a collagen scaffold. The authors compared the potential of cell-seeded and unseeded matrices to repair rotator cuff defects in an animal model. Results demonstrated that the biophysical and biochemical properties of neo-tendons improved when seeded scaffolds were used. The proper design of the scaffold, a sponge-shaped phase on a basement membrane, also appeared to play an essential role in successful tissue repair.

The interaction between MSCs and collagen, as well as collagen matrices, has also been studied. Outcomes revealed that defects treated with cell-seeded constructs exhibited better mechanical and biological properties, enabling more efficient tissue restoration than unseeded membranes. Studies have also highlighted the importance of proper cell seeding density on the scaffolding material, as this process significantly influences crucial cell-scaffold interactions, including attachment, proliferation, migration, and differentiation [30].

Collagen scaffolds can be functionalized with bioactive molecules or combined with other biopolymers [38]. Numerous studies have incorporated glycosaminoglycans, growth factors, and various cell types into collagen sponges, which preserved cell phenotype in vitro and enhanced collagen deposition at injury sites in small animal models [39].

Mechanical stimulation of the cell-colonized collagen scaffolds also plays a fundamental role in the successful tendon TE [40]. Since the engineered tissue should mimic the native tendon, the alignment of bipolar collagen fibrils in the native tendon must be recaptured in the neo-tendons. Nevertheless, cross-linking of collagen hydrogel appears to be a promising approach [32]. Mechanical loading during gel incubation is used to orient collagen fibers in the loading direction and to enhance chemical cross-linking in a magnitude-dependent manner [41]. Additionally, boundary conditions affecting high-density collagen gels appear to regulate the function of tenocytes and ligamentous fibroblasts, facilitating the development of hierarchical collagen fibers [42].

#### 4.1.2. Silk

Silk is another heavily studied natural polymer in the tendon TE. Silk fiber is typically dissolved in aqueous solution and can be reconstituted into films, mats, hydrogels, or sponges through various processing techniques [43]. These include electrospinning, freeze drying, and physical and chemical crosslinking methods. Its combination with other native polymers is also often used. Silk-based scaffolds manifest excellent mechanical, structural, and biological properties with controllable degeneration rates [44].

When applied in vivo, the tensile strength of silk fibers remains stable for up to a year and completely degrades within two years after implantation [45]. These features enable the synchronization of mechanical loads from the scaffold and the formation of a new ligament. Additionally, silk exhibits significant tenogenic and regenerative potential.

Silk has been applied in vivo to repair anterior cruciate ligaments or as a suture material in defective tendons. Chen et al. used a cell-seeded silk scaffold for in situ tendon restoration [46]. In this study, silk scaffolds with oriented and unaligned microstructures were fabricated and seeded with human stem cells derived from the periodontal ligament. To evaluate in vivo potential, seeded and unseeded matrices were applied to the defective rat Achilles tendon. Results revealed that higher tenogenic differentiation and in vivo tissue regeneration were observed within cell-seeded and aligned silk scaffolds. Moreover, macrophage polarization from M1 to M2 phenotype was also determined and modulated by colonized and oriented scaffolds.

Silk was also mixed with collagen in several studies to prepare an artificial scaffold for tendon repair. Qian et al. applied this combined matrix to defective tendons and bones in rabbits [47]. Scaffolds were prepared in 4 forms—unaligned collagen or aligned collagen scaffolds and aligned or random combined scaffolds (knitted silk/collagen). Collagen matrices were seeded with rabbit BMSCs, and their biological response was observed in vitro. Results showed good biocompatibility of the material. Elongated cell morphology was observed in the scaffold with aligned fiber orientation. On the other hand, cells plated on the random collagen scaffolds were more polygonal in shape. Compared to unaligned scaffolds, aligned ones significantly enhanced tenogenic differentiation. Combined matrices were used in an in vivo experiment where the rabbit rotator cuff was repaired. Outcomes revealed that random knitted silk/collagen scaffolds demonstrated superior regenerative potential compared to the others.

#### 4.1.3. Fibrin

Fibrin, a naturally derived biopolymer, has been extensively investigated as a scaffold for tendon TE because of its biocompatibility, biodegradability, and capacity to support cell adhesion and ECM deposition [48]. It can create a biological microenvironment that supports tenocyte proliferation, collagen synthesis, and tendon regeneration [49].

One of the main benefits of fibrin scaffolds lies in their ability to drive tenogenic differentiation by promoting the synthesis of collagen I and III, key structural components of tendons. Additionally, loading fibrin with PDGF, VEGF, or TGF-β supports cell migration and ECM remodeling, accelerating tendon regeneration [50].

However, the native fibrin scaffolds lack the mechanical strength needed to resist the high mechanical loads associated with tendons. To circumvent this shortcoming, researchers have developed composite fibrin scaffolds reinforced with materials such as collagen, silk fibroin, and synthetic polymers to enhance their durability while maintaining biological function. Moreover, 3D bioprinting has been used to produce spatially oriented fibrin scaffolds that mimic the aligned architecture of native tendons [51].

Recent work by Liao et al. [52] demonstrated the potential of tissue-engineered fibrin-based patches for repairing large rotator cuff defects. In this study, fibrin glue was combined with autologous biceps tendon particles to form a biological scaffold that supported cell proliferation, collagen synthesis, and tendon regeneration. In vitro experiments showed that fibrin glue markedly enhanced tendon stem/progenitor cell (TSPCs) viability and collagen production, both essential for functional tendon healing. Furthermore, in an animal model, they demonstrated that fibrin-based tendon patches were superior to untreated defects and fibrin glue alone in terms of tendon regeneration, collagen fibril orientation, and immune modulation. These findings confirm the clinical potential of fibrin-based tissue-engineered scaffolds and strongly support their application in tendon regenerative therapy, particularly for the treatment of rotator cuff repairs and massive tendon defects.

#### 4.1.4. Chitin

Chitin, a naturally occurring polysaccharide found in arthropod exoskeletons and fungal cell walls, has attracted considerable attention in tendon TE [53]. Chitin-based scaffolds, including chitosan derivatives, have been designed to mimic the ECM, promote tenocyte adhesion, and support tendon regeneration [54]. Moreover, chitin fibers possess high tensile strength, making them promising candidates for tendon scaffold fabrication. Reinforcement of chitin scaffolds with hydroxyapatite or collagen further enhances mechanical properties and tenocyte functionality [55].

Several studies have explored hybrid chitin scaffolds combined with alginate, silk fibroin, or synthetic polymers to improve mechanical strength while preserving biological activity. Notably, alginate–chitin scaffolds have been shown to enhance supraspinatus tendon–bone healing in vivo [55,56].

Although chitin-based scaffolds have several benefits, some limitations still need to be addressed. For instance, their high degradation rate necessitates modifications to increase scaffold stability through crosslinking [57]. Moreover, their reduced bioactivity in comparison to other biomaterials necessitates the functionalization of growth factors. Furthermore, these scaffolds often require reinforcement with composite materials to enhance their mechanical properties for load-bearing tendon applications [55].

#### 4.1.5. Decellularized Tissues

Scaffolds derived from decellularized tissues have been applied in pre-clinical and clinical trials [58]. Processing these matrices must involve the precise removal of cells, toxins, and lipids while retaining their collagenous structure [59]. The acellular dermis, tendon, amniotic membrane, small intestinal submucosa, and pericardium are examples of biological scaffolds that were also approved by the FDA for tendon repair (e.g., Arthroflex^®^ (Arthrex, Inc., Naples, FL, USA), Dermaspan™ (Zimmer Biomet, Warsaw, IN, USA), etc.) [60].

The scaffolds mentioned above were primarily used to restore Achilles tendon ruptures and rotator cuff tears. According to the results, they present an attractive material for tendon regeneration. In addition, a novel approach described the application of scaffolds derived from the ECM. Findings indicated that such matrices were capable of recruiting progenitor cells and regulatory molecules from the site of interest, thereby triggering the repair processes. This was also investigated by a study by Cui et al. [61]. They utilized tendon-derived stem cells to form an ECM, which was then combined with a decellularized bovine tendon sheet to create a scaffold possessing enhanced biomechanical and biochemical properties.

BMSCs were utilized to assess their biological properties, including attachment, proliferation, migration, and differentiation. Matrices were also applied in vivo to repair damaged Achilles tendons. Outcomes revealed that a scaffold based on an ECM and a decellularized tendon sheet showed superior biological, mechanical, and regenerative properties compared to a pure decellularized tendon sheet. Moreover, results also demonstrated that combined scaffolds recruited endogenous stem cells.

Lun et al. [62] showed that modifications of decellularized tendon membranes, such as the incorporation of MnO_2_, significantly enhance mechanical stability, bioactivity, and resistance to degradation. Their findings underscore the significance of crosslinking and composite reinforcement strategies in enhancing decellularized tissue scaffolds for tendon repair applications.

### 4.2. Synthetic Scaffolds

Synthetic scaffolds are derived from synthetic polymers and can be further categorized into biodegradable and non-biodegradable types [63]. Poly(ε-caprolactone) (PCL), poly-lactic acid (PLA), polyglycolic acid (PGA), and their copolymers are just a few examples used in scaffold fabrication [64,65]. Unlike natural matrices, synthetic scaffolds display better mechanical, physical, and structural properties and can be precisely designed or modified (Table 1) [66]. They also allow improved control over degradation. Moreover, they can be easily sterilized, so the risk of pathogen transmission is lower when compared to the natural matrices. Several studies have also confirmed their favorable biological behavior, creating an appropriate environment for cell attachment, proliferation, and differentiation [64]. Synthetic scaffolds also have several drawbacks. Most do not naturally occur in living organisms, which increases the risk of an inadequate immune response. Additionally, their utilization can involve dissolving them in chemical solvents, which may alter their biocompatibility. Due to the hydrophobic nature of these materials, the cellular response may deteriorate in terms of cell adherence, proliferation rate, and phenotypic changes [67]. Several approaches that improve the disadvantages above are physical or chemical surface modification. These techniques involve immobilizing bioactive compounds and micro- or nanopatterning to create structured cellular arrays, thereby influencing cell behavior [68].

Importantly, attention must also be focused on the by-products released during their degradation, which may cause adverse effects. However, this issue can be addressed by in vitro testing of the material’s cytotoxicity.

#### 4.2.1. Polylactic Acid

Poly(L-lactic) acid, the L enantiomer of PLA, PLLA, exhibits excellent mechanical properties and biocompatibility, making it more suitable for load-bearing applications, particularly in tendon, ligament, and tendon/ligament-bone interface scaffolds [69]. This polyester occurs naturally in the body, and its degradation produces lactic acid, a metabolite readily processed without eliciting an immune response [70]. However, the biopolymer also exhibits several drawbacks, including a slow degradation rate, limited ductility, and hydrophobicity [71].

PLLA scaffolds have primarily been fabricated using electrospinning, braiding, and knitting techniques. Electrospun PLLA nanofibers with mechanical, structural, and compositional gradients were designed to replicate the bone–tendon interface. Knitted PLLA fibers have also been employed to regenerate the medial collateral ligament (MCL) in a rabbit model, resulting in type I collagen expression and fibrocartilage formation [72].

The current study conducted by Stodolak-Zych et al. [73] examined the effect of PLA-based implants on anterior cruciate ligament healing when used as a supportive component in autologous grafts. When implanted into defective knee joints of sheep, suitable biomechanical parameters were observed, which were also reflected in a lower joint failure force. In addition, better engraftment in the tendon–bone surface was also determined. In another study, PLLA electrospun nanofibers were successfully utilized to construct hierarchical scaffolds for the regeneration of tendons and ligaments. Hierarchical matrices were constructed by bundling fibers and wrapping them within a nanofibrous sheath, recreating the compact architecture of native tendon. These scaffolds demonstrated tensile strength appropriate for tendon replacement [74]. Knitted PLA scaffolds seeded with MSCs showed increased expression of collagen I, tenascin-C, decorin, integrins, and matrix MMPs, an effect further promoted by growth factor addition [75]. Enhancing PLA performance typically involves blending or copolymerization with lactic acid, glycolide, or ε-caprolactone.

#### 4.2.2. Polyglycolic Acid

PGA exhibits higher strength and hydrophilicity than PLA, and its self-reinforced form is markedly stiffer than other clinically used biopolymers, making it suitable for the bone side of tendon/ligament–bone interface scaffolds [76]. However, PGA undergoes rapid degradation, and the accumulation of glycolic acid as a by-product can locally lower pH and cause tissue damage [77]. For instance, PGA braided scaffolds seeded with rabbit anterior cruciate ligament cells showed poor ECM formation due to scaffold degradation within one week [78].

#### 4.2.3. Poly (Lactic-co-Glycolic) Acid

Compared with its homopolymers PLA and PGA, poly(lactide-co-glycolide) (PLGA) offers superior control over degradation rate, which can be finely tuned by adjusting the monomer ratio to modulate crystallinity and hydrophilicity [79]. However, pure PLGA exhibits limited hydrophilicity, suboptimal mechanical strength, and low bioactivity, constraining its direct clinical use. Consequently, in bone and tendon/ligament regeneration, PLGA is often blended with other biomaterials, such as aliphatic polyesters like PLA [80]. In tendon TE, PLGA is commonly fabricated by electrospinning; fibroblast-seeded PLGA scaffolds have demonstrated cell alignment, distribution, and ECM deposition corresponding to fiber orientation, thereby closely replicating native tendon architecture. Moreover, the mechanical properties were improved by applying cyclic mechanical strain [81]. Electrospun PLGA nanofibers containing basic fibroblast growth factor (bFGF) were utilized to mimic the native ECM structures, thereby enhancing the proliferation and attachment of mesenchymal progenitor cells [82]. Bone morphogenetic protein 2 was loaded into PLGA and used for reconstruction after anterior cruciate ligament rupture in rats. As confirmed by in vitro and in vivo experiments, the combination of this synthetic scaffolding and growth factor effectively induces the formation of blood vessels. It effectively regulates the inflammation and regeneration of tendons and bones [83].

PLGA nanofibers have also been electrospun with aligned-to-random orientations to mimic the native tendon–bone interface, introducing gradients in mechanical and structural properties. The highly aligned nanofibers enhanced tensile strength and promoted cell alignment along the longitudinal axis of the fibrous architecture [84].

In an in vitro study, braided PLGA scaffolds provided appropriate viscoelasticity and mechanical stability, with large inter-fiber pores that facilitated collagen infiltration throughout the structure. Such constructs can also serve as delivery systems for growth factors or bioactive agents to enhance tissue repair [85]. bFGF-loaded PLGA scaffolds improved MSC proliferation and tenogenic differentiation, marked by elevated collagen type I expression, while BMSCs seeding under static culture promoted collagen deposition both in vitro and in vivo [86].

#### 4.2.4. Polyurethanes

Polyurethanes represent a heterogeneous and highly versatile class of polymers whose physical and mechanical properties depend on the specific isocyanates and polyols used in synthesis [87]. Their main advantage lies in the ability to tailor mechanical strength, elasticity, porosity, and degradation behavior to meet scaffold design requirements [88]. Various fabrication approaches have been explored for polyurethane-based scaffolds. For instance, a polycarbonate–polyurethane patch without cell seeding successfully repaired a supraspinatus tendon defect, promoting significant tissue growth [89]. When seeded with fibroblasts and exposed to cyclic strain, such patches exhibited an increased elastic modulus compared with non-stimulated controls. Another study assessing fibroblast-seeded polyurethane scaffolds under cyclic loading demonstrated enhanced cell proliferation and matrix accumulation [90]. Electrospun polyurethane scaffolds also showed excellent biocompatibility, supporting cell attachment, maturation, and collagen deposition [91]. However, a key limitation of some polyurethane formulations is the potential release of cytotoxic degradation by-products [92].

#### 4.2.5. Poly (ε-Caprolactone)

PCL is a semi-crystalline aliphatic polyester that is bioresorbable and non-toxic to cells and living tissues, making it widely used in TE [93]. PCL is a ductile polymer characterized by relatively low tensile strength and modulus but high elongation at break. Its degradation rate is approximately three times slower than that of PLA, which may limit its application in biodegradable tendon and ligament scaffolds [94]. To enhance mechanical performance, hydrophilicity, and biodegradability, PCL has been blended or copolymerized with varying ratios of PLA [95]. For example, fibrous PCL scaffolds were fabricated by co-electrospinning aligned microfibers and random nanofibers of PCL to create a multiscale hybrid bilayer scaffold coated with a chitosan-hyaluronic acid hydrogel for ligament regeneration [96]. To investigate the influence of scaffold architecture on tendon repair, PCL scaffolds were electrospun into various configurations, including 2D random sheets, 2D aligned sheets, and 3D bundles. The tensile strength of the 3D constructs did not fully replicate that of native tendon tissue but could be improved through braiding techniques and material engineering. In another study, a tubular, multilayered PCL scaffold was fabricated via electrohydrodynamic jet printing [97]. This design incorporated thick fibers as a supportive framework and thin fibers as alignment cues for cells. Human tenocytes cultured on this scaffold exhibited longitudinal alignment along the fiber bundles, accompanied by collagen deposition.

Gwiazda et al. [98] employed melt electrowetting to produce PCL microfibrous scaffolds intended for bone–ligament–bone regeneration. This approach allowed precise control over microstructure and uniform cell distribution. In a more recent study, Wang et al. [99] engineered a PCL scaffold with a gradient distribution of hydroxyapatite (HA) nanoparticles. The resulting construct successfully mimicked the native tendon–bone interface, enhancing biomechanical performance and promoting cellular integration—features that make PCL–HA gradient scaffolds a promising strategy for tendon and ligament TE. The material’s attractiveness for tenocytes can be increased by coating it with collagen.

### 4.3. Hybrid Composites

Synthetic and natural biopolymers have already been employed in tendon TE applications, and both have demonstrated their advantages and disadvantages. The main advantage of synthetic biopolymers is their mechanical strength, whereas natural biopolymers excel in their ability to bio-mimic the natural characteristics of the ECM [100]. For designing scaffolds intended for tendon TE, synthetic materials are crucial components due to their resistance and mechanical properties. Despite offering adequate mechanical support, these materials do not fully support biological regeneration [101]. They serve primarily as structural reinforcements for tendon defects but exhibit limited bioactivity. To address this limitation, efforts have focused on improving biocompatibility by combining with hydrophilic, biocompatible hydrogels. In this sense, the aim is to achieve a cooperation that enables the creation of scaffolds with good structural integrity and high biocompatibility [102]. Therefore, ongoing research is investigating the combination of materials that can replicate the mechanical characteristics of tendons and address efficacy issues. Most hybrid scaffolds have been prepared by electrospinning because they present a low-cost and straightforward method. In this context, PCL, PLA, and PLGA are the most used synthetic biopolymers blended with natural polymers, primarily collagen, to improve the biocompatibility and bio-functionality of electrospun scaffolds for tendon repair [82,103].

Sensini et al. [104] fabricated electrospun PLLA/collagen-aligned 3-D nanofibrous scaffolds in bundles. The engineered matrix also fulfilled biological requirements when seeded with fibroblasts. Moreover, good mechanical properties were maintained after prolonged immersion in PBS.

PLLA fibers have also been braided to fabricate tendon scaffolds with excellent mechanical properties and subsequently combined with natural biopolymers to enhance biological performance [78]. Kimura et al. reported a PLLA braided fiber scaffold integrated with a collagen membrane designed for the sustained release of bFGF [105]. This composite scaffold provided both mechanical support and biochemical cues, facilitating regeneration of the bone–anterior cruciate ligament–bone interface in rabbit models. Results revealed improved mechanical properties of regenerated tendons due to increased cell migration within the scaffold. Higher collagen deposition was also detected. Moreover, the local release of bFGF also seemed to have a significant effect on bone regeneration.

Conversely, PLLA blended with collagen and electrospun into fiber bundles was used to reconstruct the fascicular architecture of the native Achilles tendon [106]. While collagen incorporation enhanced the attachment and proliferation of human tenocytes, the electrospun matrices exhibited markedly reduced mechanical strength in vitro, primarily due to the rapid degradation of the natural polymer.

To promote osteogenic activity during tendon–bone interface regeneration, calcium phosphate silicate (CPS) ceramic was incorporated into PLLA. A study conducted by Guo et al. [107] described how PLLA/CPS membranes enhanced collagen orientation, osteogenesis, and chondrogenesis after implantation in rabbit models.

In another study, silk fibroin was combined with poly(p-dioxanone) to fabricate a wavy nanofibrous scaffold via electrospinning technology and thermal ethanol treatment. The outcome of the study showed that the mass ratio of used polymers could regulate specific structural and mechanical properties. From a biological perspective, increasing the silk ratio enhanced the affinity of human tenocytes to the scaffold [108]. In vivo studies have demonstrated the favorable biocompatibility of the composite material compared to pure poly(p-dioxanone) membranes [109]. Moreover, loading with growth factors and mechanical stimulation significantly enhanced the tenogenic differentiation of seeded MSCs [108].

A PLGA/wool keratin composite membrane was fabricated by emulsion electrospinning and subsequently loaded with bFGF to improve the in situ regeneration [110]. Moreover, bFGF was also combined with dextran to ensure its better encapsulation. Results revealed the optimal concentration of bFGF, which provides the best environment for cellular response. Another composite scaffold for potential utilization in tendon or ligament TE was prepared using a cell-loaded alginate gel that encapsulated a knitted structure of PLGA [111]. The authors of this research investigated whether alginate could serve as a cell carrier and deliver cells to the site of the defect. The knitted structure should have provided mechanical strength to the composite scaffold. When applied in vivo, defective tendons treated with cell-seeded constructs demonstrated improved elasticity, enhanced cell proliferation, and enhanced vascular supply. In a recent study, Wang et al. [112] analyzed PLA/PGA hybrid yarn-based tubular mesh developed through melt-spinning and braiding, which exhibited strong mechanical properties, allowing it to sustain natural tendon strain. They demonstrate that full mechanical loading on the scaffold efficiently induces tendon regeneration, as evidenced by parallel-aligned collagen fibers and tenocytes, which mimic the native tendon structure. Additionally, transmission electron microscopy revealed that mechanical strain enhanced collagen fibril development, resulting in increased fibril diameter and improved mechanical properties. Notably, the synergistic effect of mechanical loading and hyaluronic acid modification further promoted the tenogenic differentiation of infiltrated fibroblasts, thereby enhancing the efficacy of the scaffold.

All presented studies emphasize that polymers, in general, play a crucial role in TE and regenerative medicine. Although numerous experiments have been carried out on animal models, clinical trials are still lacking, thereby delaying the translation of novel approaches into practical medicine.

### 4.4. Additive Manufacturing

**Figure 5:**
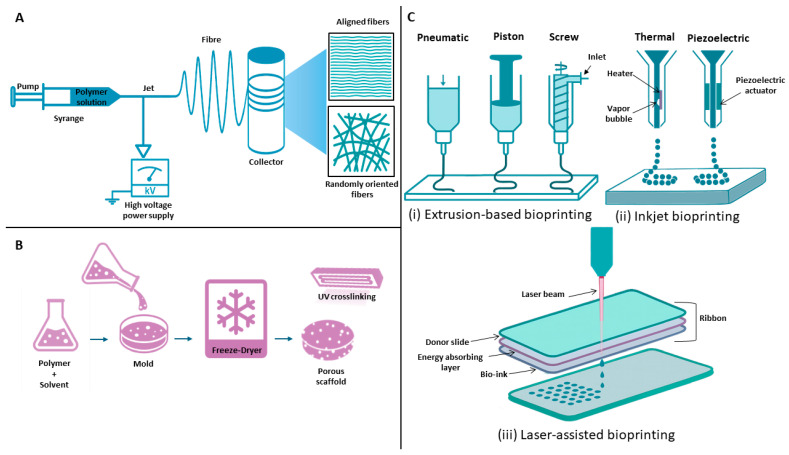
Schematic illustration of fabrication techniques used in preparation of biomaterial scaffolds. (**A**) Electrospinning process for generating nanofibrous structures with either aligned or randomly oriented fibers. (**B**) Freeze-drying method for producing porous scaffolds from polymer solution followed by UV crosslinking. (**C**) Overview of bioprinting techniques (i) extrusion-based, (ii) inkjet, and (iii) laser-assisted bioprinting, demonstrating different mechanisms for bioink deposition. Figure was drawn by authors in Microsoft PowerPoint and GIMP.

Additive manufacturing (AM) has transformed numerous biomedical fields by enabling the fabrication of complex, patient-specific structures with high reproducibility and precision. Although conventional technological approaches such as electrospinning and freeze-drying continue to play a significant role in tendon TE, AM facilitates the creation of more precise bioengineered scaffolds, medical implants, and tissue constructs customized for individual patients (Figure 5) [113].

Unlike conventional subtractive methods that remove material, AM constructs objects layer by layer, providing superior control over geometry, porosity, and mechanical properties (Table 2) [114].

In TE, a principal application of AM is the fabrication of scaffolds that serve as temporary templates enabling cell attachment, proliferation, and their differentiation. Using biomaterials that mimic the native ECM, AM enables precise production of porous structures that promote vascularization and integration with host tissue [123]. Given its distinct benefits, AM has emerged as a valuable tool in tendon TE.

#### 3D Bioprinting

3D printing enables the layer-by-layer fabrication of bioengineered structures with defined geometries using printable materials, or “inks.” In TE, this technology is applied in two primary ways: conventional 3D printing technique that engineers acellular scaffolds from biomaterial inks, which are later seeded with cells and growth factors, or as 3D bioprinting, which directly incorporates living cells and bioactive molecules into the printing ink. These inks are specifically named bioinks—composites of biomaterials and cells designed to replicate the ECM microenvironment [124]. Bioinks support cell adhesion, proliferation, and differentiation, allowing the generation of constructs that closely mimic the morphology and functionality of native tissues [125]. Key advantages of 3D printing include precise geometric control (morphology, pore size, porosity), automation potential, and compatibility with a broad range of materials. Bioactive agents such as drugs, proteins, and living cells can be incorporated, and gradients of cell density can be designed to emulate native tissue organization [126]. Although printing processes may induce mechanical stress and inadequate vascularization can hinder tissue survival current research is focused on developing strategies to overcome these limitations [127].

3D bioprinting encompasses several techniques, including inkjet-based bioprinting, extrusion-based bioprinting, and laser-assisted bioprinting, which differ in their printing mechanisms, resolution, and compatibility with various bioinks [128].

Inkjet bioprinting is a non-contact technique that generates bioink droplets under pressure [129]. The two principal mechanisms are thermal and piezoelectric actuation. In thermal inkjet printing, a rapid electric pulse heats a resistive element, creating a transient vapor bubble (~300 °C for a few microseconds). The resulting pressure ejects a bioink droplet through the nozzle. The short heating duration minimizes cellular damage [93,129]. Inkjet printing offers high resolution, speed, and cost efficiency, but it is limited to low-viscosity or aqueous bioinks to avoid nozzle clogging [124].

Extrusion bioprinting deposits continuous filaments of bioink using pneumatic pressure or mechanical force. This technique accommodates high-viscosity materials and enables the creation of mechanically robust constructs. Extrusion printing offers an excellent price-to-performance ratio, simplicity, and compatibility with diverse biomaterials, making it the most widely used method [130]. Compared to inkjet printing, it allows higher cell densities but at the cost of lower resolution. High pressure and viscosity can reduce cell viability, which may range from 45% to 90% depending on ink composition, nozzle diameter, and flow parameters [129]. Achieving an optimal balance between bioink viscosity, printability, and cell survival remains a major challenge [127].

Laser-assisted bioprinting (LAB) employs a focused laser pulse to deposit droplets of bioink without physical contact, eliminating nozzle clogging and allowing use of high-viscosity materials [131]. The system consists of two layers: an upper donor layer (energy-absorbing) and a lower layer containing bioink. Localized laser irradiation vaporizes the donor layer, forming a high-pressure bubble that propels a droplet toward the receiving substrate. By precise substrate movement, 3D structures are assembled layer by layer [130]. LAB offers exceptional spatial accuracy, high resolution, and minimal mechanical stress on cells. However, localized thermal and mechanical effects may slightly reduce cell viability. The technique also faces challenges related to high equipment costs, complex calibration, and the optimization of laser energy, pulse duration, and bioink composition. LAB remains an emerging, yet highly promising, technology for fabricating functional tissue constructs [132].

Silva et al. utilized 3D printing approach to develop a biocompatible PLA/graphite-based scaffold for tendon TE [115]. Functionalization of graphite with silver particles conferred antimicrobial properties to the matrix. Fibroblasts and human tendon-derived cells were used to evaluate the scaffold’s biological performance, revealing good biocompatibility and enhanced expression of tendon-associated biomarkers, suggesting that the scaffold promotes tendon-specific biological processes.

Filippi et al. developed a 3D-bioprinted muscle-tendon unit that integrates skeletal muscle tissue with tendon-mimicking fibroblast-laden anchors, replicating the native myotendinous junction [116]. Using multimaterial extrusion-based 3D bioprinting, they produced interdigitated interfaces and stiffness gradients that improved mechanical stability and force transfer. The bioprinted constructs generated contractile forces up to 350 µN and maintained functional activity for over three months, outperforming previous bioactuator systems. Computational modeling was used to optimize muscle geometry and enhance deformation and force generation. This work demonstrates how 3D bioprinting enables the fabrication of biomimetic, mechanically optimized musculoskeletal bioactuators with improved efficiency and durability for biomedical and robotic applications.

Using 3D printing, Li et al. fabricated an injectable platelet-rich plasma hydrogel incorporating tendon-derived stem cells, demonstrating the potential of AM to precisely control scaffold architecture and composition for tendon regeneration [117]. The printed hydrogel provided a favorable microenvironment for tendon-derived stem cells and downregulated the PI3K–AKT signaling pathway, which is typically overactivated in chronic tendon pathologies.

Although only a few representative studies are discussed here, they clearly demonstrate the importance of 3D printing in tendonTE, where the detailed scaffold design facilitates the formation of tendon-like tissues in vitro and contributes to inflammation modulation and regeneration enhancement.

### 4.5. Electrospinning

Electrospinning is a versatile and scalable technique for fabricating micro- and nanofibrous scaffolds with high surface area and porosity [133]. By manipulating process parameters and collector design, fibers can be produced with random or aligned orientation, the latter being particularly advantageous for tendon applications [134,135]. The method offers simplicity, tunability, and compatibility with various biomaterials, yielding scaffolds with interconnected pores and good mechanical adaptability [136]. However, its limited control over pore architecture and restricted cell infiltration present ongoing challenges for constructing large three-dimensional tissue-engineered structures [137]. Nevertheless, electrospinning remains a key engineering technique in TEand has been extensively investigated for tendon applications.

A study conducted by Olvera et al. optimized the electrospinning of PCL by adjusting the rotational speed of the collecting mandrel to minimize fiber fusion [118]. The resulting unfused fibers enabled the formation of highly porous (up to 95%) 3D scaffolds. Achieved architecture promoted rapid MSC infiltration and differentiation toward tendon and fibrocartilage lineages. Overall, the approach provides a simple strategy to fabricate porous, mechanically robust electrospun constructs suitable for tendon as well as other musculoskeletal tissue regeneration. Guner et al. were evaluating, how the fiber orientation within the dual-phase scaffold influenced biological behavior of seeded cells [119]. Rotary jet spinning was applied to produce scaffolds’shell based on aligned fibers of PCL. Core consisted of randomly oriented PCL, or PCL/gelatine fibers and was engineered via wet electrospinning. Results revealed that aligned outer shell guided cell elongation and orientation, promoting organized cell growth and ECM deposition characteristic of healthy tendon tissue. In contrast, the randomly oriented inner core enhanced initial cell attachment and viability, resembling the early repair phase of healing. When treated with growth differentiation factor 5 (GDF-5), the scaffold further improved tenogenic differentiation, collagen type III production, and cell infiltration. Overall, the scaffold’s fiber alignment played a critical role in directing cell behavior and supporting the regeneration of tendon-like tissue with high mechanical integrity. The fabrication of hierarchically structured scaffolds with controlled fiber orientation remains a major focus in tendon TE, as recent studies have underscored its pivotal role in promoting effective tendon repair [138,139,140,141,142]. Moreover, recent study carried out by Yang et al. introduced a dual-functional biomimetic tendon sheath designed to both promote tendon healing and prevent peritendinous adhesion [143]. The sheath was fabricated via coaxial electrospinning, creating a nanofibrous membrane with a celecoxib-loaded PCL core and a gelatin methacryloyl (GelMA) shell. The GelMA shell provided a favorable microenvironment that enhanced tenogenic differentiation of TSPCs, while the controlled release of celecoxib effectively reduced inflammation. In vivo experiments on tendon injury models showed significant decreases in adhesion and improved structural repair. Overall, this coaxial nanofibrous membrane demonstrates strong potential as a dual-action biomaterial for improving tendon regeneration and minimizing post-surgical complications.

### 4.6. Freeze-Drying

Lyophilization (freeze-drying) is a simple fabrication method that preserves the bioactivity of natural polymers. The process involves freezing a polymer solution followed by sublimation of ice under vacuum, yielding a porous, interconnected 3D scaffold suitable for TE applications [144]. Freeze-dried scaffolds possess tunable porosity and aligned architectures favorable also for tendon regeneration [145]. Natural polymers such as collagen, gelatin, and silk fibroin have been already proccessed by this technique providing engineering of biocompatible, ECM-like scaffodls [146]. The main limitation of freeze-drying is the low mechanical strength of the resulting scaffolds [147]. Reinforcement approaches include chemical crosslinking, blending with synthetic polymers (e.g., PCL), and post-fabrication mechanical conditioning [148]. The study by Basile et al. investigated the use of freeze-dried tendon allografts as scaffolds for gene delivery in tendon TE [121]. The authors demonstrated that freeze drying preserves the mechanical properties of tendon grafts while allowing efficient loading with recombinant adeno-associated viral vectors carrying the *GDF5* gene. The *GDF5* gene encodes a protein that plays a crucial role in tendon and ligament development, repair, and remodeling by promoting cell proliferation, differentiation, and ECM organization. These freeze-dried allografts successfully mediated localized and transient gene expression in vivo, enhancing wound healing and improving joint flexion compared to controls. Importantly, the process produced biocompatible, acellular scaffolds with strong hydrophilic properties and a long shelf life. Overall, this work highlights freeze drying as a promising method for creating therapeutically enriched tendon grafts for regenerative applications. Another study examined how the freeze-drying process can be used to control pore size in collagen–glycosaminoglycan scaffolds for TE applications [122]. By varying the freezing temperature and introducing an annealing step, researchers were able to tailor scaffold structures for different applications. Lower freezing temperatures produced smaller pores, while annealing during freeze drying increased pore size by about 40%. The optimized method generated scaffolds with mean pore sizes ranging from 85 to 325 μm, which demonstrated significant improvement over previous limits. These findings are highly relevant to tendon TE, where controlling scaffold porosity is essential for guiding cell infiltration, collagen alignment, and nutrient diffusion. These key factors are crucial for engineering functional, load-bearing tendon constructs.

Presented results highlight the importance of achieving proper scaffold architecture in order to effectively repair damaged tendon tissue. These properties can be achieved by specific AM as well as electrospinning or freeze-drying technique. Moreover, subsequent modification can enhance biological behavior of scaffold as well. Although many experimental studies have achieved promising results, translation into clinical medicine is still lacking.

## 5. Discussion

Presented manuscript highlights recent progress and persistent challenges in the field of tendon TE. Conventional approaches to tendon injury management, including surgical repair and conservative therapy, are often limited by scar formation, poor vascularization, and incomplete restoration of mechanical properties. Emerging strategies in TE and regenerative medicine offer potential solutions to these problems.

**Figure 6:**
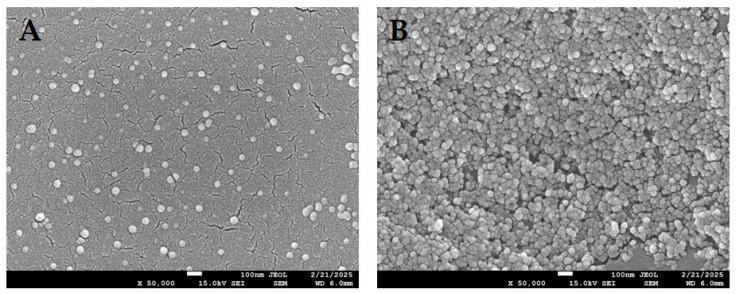
Exosomes. Scanning electron microscopy (SEM) depicting the morphology of exosomes derived from mesenchymal stem cells. (**A**): exosomes exhibiting characteristic round morpohology; (**B**): concentrated exosomes forming large cluster. All SEM micrographs were acquired using a JEOL 7500F scanning electron microscope (JEOL Ltd., Japan) operated by Martina Culenova.

From a cellular perspective, tenocytes remain the most physiologically relevant population for tendon regeneration, yet their limited availability and tendency to dedifferentiate in vitro restrict their applicability [149]. However, MSCs provide an adequate cellular alternative thanks to their excellent differentiation capacity and paracrine signaling activity. Increasing attention is directed toward cell-free approaches, such as EVs or MSCs’ secretome, which could mitigate immunogenic risks and simplify regulatory approval [17,18,19,20,21,22]. Exosomes are part of EVs with average size of 100 nm (Figure 6). Depending on their origin, they carry many biologically active molecules such as nucleic acids, lipids, metabolic or cell surface antigens [150]. A recent study that investigated the healing potential of exosomes derived from tenocytes and MSCs also determined that their origin significantly affect their biological activity [151]. In this case, tenocyte-derived exosomes enhanced osteoclast differentiation and decreased the synthesis of pro-inflammatory cytokines. Moreover, macrophage-driven response from tenocyte-derived exosomes resulted in higher tenocyte migration, and matrix degradation was suppressed. Although MSCs-derived exosomes also activated local regenerative pathways, the findings indicate that tenocyte-derived exosomes may exert a more favorable healing effect on tendon repair.

Scaffold design is another critical pillar of tendon TE. Natural polymers such as collagen, silk, fibrin, and decellularized tissues provide excellent biocompatibility but often lack sufficient mechanical integrity. In contrast, synthetic scaffolds (PLA, PGA, PLGA, PCL, polyurethanes) offer tunable mechanical and degradation profiles but may suffer from reduced biocompatibility or unfavorable by-products. Hybrid scaffolds that combine natural and synthetic constituents are increasingly being explored to achieve both structural stability and biological functionality [25,64]. To achieve the best biological response of the scaffold, a proper manufacturing technique must be chosen. Currently, electrospinning, freeze-drying, and particularly 3D bioprinting are revolutionizing the field by enabling precise control over scaffold architecture, fiber alignment, and biochemical gradients. In the context of the tendon TE, these advances facilitate the recreation of hierarchical tendon structures and may improve tendon-to-bone integration. Nonetheless, several barriers remain before clinical translation can be achieved. Chief among these is the establishment of robust vascular networks, the maintenance of long-term mechanical stability under physiological loading, and the prevention of adverse immune responses.

The challenges mentioned could be overcome by acquiring knowledge from the emerging field of regenerative immunoengineering. This discipline utilizes biomaterial surface geometrics and inner architecture to minimize foreign body reaction and maximize positive biological and immunological response in vivo [152].

Hydrogels have drawn attention, particularly for good results in tendon regeneration in vitro and in vivo [153,154,155]. Moreover, specific hydrogel based on polyglutamic acid and polylysine had been developed to regulate inflammatory response in damaged tendon [156]. Hydrogel was enriched with rhein, 1-ethyl-(3-dimethylaminopropyl) carbodiimide and N-hydroxysuccinimide. This formula proved to be effective in inhibition of M1 macrophage polarization. Additionally, expression of pro-inflammatory cytokines had been reduced as well.

Scaffold loading with small bioactive molecules, peptides, growth factors, anti-inflammatory drugs, antibiotics, or nucleic acid therapeutics (plasmid DNA, siRNA, viral vectors) holds promising approach towards modulation of the healing environment [157]. Hydrogels and electrospun fibers are commonly used carriers. Release of the agents is controlled by diffusion, degradation, or stimulus-triggered mechanisms. Controlled delivery allows high local concentrations with reduced systemic exposure and can be staged (early anti-inflammatory agents, later teno-inductive factors) to match biological as well as pathological processes [158].

Scaffolds can also be loaded with different types of cells. In the context of tendon TE, tenocytes, TSPCs, or MSCs are typically seeded into scaffolds [159]. Cell seeding provides a source of matrix-producing cells and paracrine factors that modulate inflammation and host repair. Seeding approaches range from pre-culture to form cell-rich grafts, to in situ cell delivery (injectable cell-laden hydrogels). Evidence shows that aligned scaffold architecture together with mechanical stimulation favors tenogenic differentiation of seeded progenitors—a key principle exploited by many recent designs [143].

Recent advances in gradient scaffold design have shown great promise for regenerating the tendon-to-bone interface, one of the most challenging regions in musculoskeletal repair [160]. This interface features a gradual transition from soft, collagen-rich tendon tissue to hard, mineralized bone, and replicating this complex gradient is key to achieving functional integration. Studies utilizing nano- and microstructured gradient scaffolds have demonstrated their ability to guide spatial cell differentiation and matrix deposition, promoting the formation of zonal tissue organization similar to the native enthesis. Such scaffolds not only enhance mechanical strength and load transfer but also reduce the formation of fibrous scar tissue that often compromises repair outcomes. Therefore, incorporating gradient architectures, which can be potentially achieved through controlled freeze-drying or electrospinning, offers a biomimetic strategy to improve tendon-to-bone healing and advance the clinical translation of tendon TE approaches.

Novel approach is also presented by advanced scaffolding material with the ability to response to the stimuli obtained from the environment surrounding damaged tendon [161]. These stimuli-responsive scaffolds are able to change their properties or release load in response to both endogenous (pH, enzyme activity, local mechanical strain) or exogenous (temperature, magnetic fields, light, ultrasound, electrical fields) cues. These responses can be utilized to provide on-demand and stage-specific delivery of bioactive molecules, to apply mechanical cues to cells, and also alter scaffold mechanics or porosity to promote cell infiltration and matrix remodeling during healing process [162]. For tendon applications, the ability to match varying stages during tendon regeneration, e.g., anti-inflammatory action in the acute phase followed by teno-inductive cues during remodeling, is particularly attractive.

Presented results highlight the importance of TE in effective repair of damaged tendon. Current strategies focus on cell-based and cell-free approaches, including the use of tenocytes, MSCs, and exosomes, which enhance regeneration while minimizing immunogenicity. Advances in scaffold design—particularly hybrid, gradient, hydrogel-based, and stimuli-responsive scaffolds—enable precise control of biochemical cues, drug or cell delivery, and structural mimicry of native tendon tissue. Despite these advancements, key challenges such as vascularization, mechanical stability, and immune compatibility must be resolved to achieve successful clinical translation.

## 6. Conclusions and Future Direction

Tendon TE has advanced significantly in developing biologically driven scaffolds, refining cell sources, and incorporating bioactive molecules to mimic the complex structure and function of native tendon tissue. While significant advances have been made in scaffold fabrication methods—namely, electrospinning, freeze-drying, and 3D bioprinting—there remains a pressing need to translate these findings into clinically meaningful solutions. The use of medical-grade polymers, already approved for direct medical applications, may facilitate progress in this field. However, current evidence suggests that without additional modification by biologically active agents—such as stem cells, growth factors, or cytokines—the therapeutic effect may not reach the expected magnitude. Each such biological modification carries certain risks for the patient. Therefore, selected products must advance into preclinical testing as soon as possible, since without these results, research cannot progress toward the real implementation of tissue-engineered products in clinical medicine, particularly in the management of tendon injuries.

Future studies should focus on incorporating vascularization approaches, such as pre-vascularized constructs or the delivery of angiogenic factors, to ensure long-term graft survival and remodeling. Additionally, mechanical conditioning protocols in bioreactor systems require optimization to simulate physiological loading and direct tenogenic differentiation more efficiently. Advances in gradient scaffolds, particularly those that mimic the transitional interface between tendon and bone (enthesis), hold promise for the functional repair of complex injuries.

The incorporation of AM technologies with bioactive hydrogels and cell-laden bioinks enables the construction of patient-specific, zonally organized tendon grafts. Cell-free approaches, which involve EVs or secretomes, can also mitigate immunogenic risks and simplify regulatory pathways.

Interdisciplinary collaboration between tissue engineers, biomaterials researchers, clinicians, and regulatory authorities will be essential to bridge translational divides. Carefully designed long-term in vivo experiments and clinical trials must be conducted to establish the safety, efficacy, and durability of engineered constructs. Future therapeutic advances will depend on the integration of biomaterial innovation with mechanobiology insights and scalable engineering platforms, enabling the fabrication of functionally anisotropic constructs that restore tendon integrity and load-bearing capacity in clinical settings.

Another important consideration is the economic aspect. Because these strategies integrate the latest advances in biology, materials science, and engineering, the final product will likely be costly. Nevertheless, given that regenerative medicine represents the future of therapy, the investment appears justified.

## Figures and Tables

**Table 1:** Comparison of natural and synthetic polymers for tendon TE.

Category	Polymer	Properties	Advantages	Limitations	Applications/Notes
Natural	Collagen	Biocompatible, biodegradable, porous, low immunogenicity	Excellent cell attachment, bioactivity, ECM mimicry	Low mechanical strength, rapid degradation when denatured	Used in seeded scaffolds, often cross-linked or combined with silk/synthetics
	Silk	Strong, flexible, biocompatible	Long degradation time, supports cell differentiation	May induce immune response, batch variability	Used in ACL and tendon repair, often aligned for better results
	Fibrin	Biodegradable, supports cell adhesion & ECM synthesis	Bioactive, growth factor carrier	Weak mechanical properties	Modified with collagen/silk, used in tendon patches
	Chitin/Chitosan	Strong, polysaccharide-based	Promotes tenocyte adhesion, ECM mimicry	High degradation, lower bioactivity	Combined with alginate/synthetics, needs crosslinking
	Decellularized Tissues	ECM-based structure	Native architecture, low immunogenicity	Source limitations, processing complexity	FDA-approved (e.g., Arthroflex^®^), supports endogenous repair
Synthetic	PLLA (PLA)	Biocompatible, slow degradation, good strength	Supports load-bearing, tunable properties	Poor ductility, hydrophobic	Used in electrospun/braided scaffolds, often mixed with collagen or CPS
	PGA	High strength, hydrophilic	Fast degradation	Risk of tissue damage from glycolic acid	Used in ACL regeneration, needs controlled degradation
	PLGA	Copolymer of PLA & PGA, tunable degradation	Versatile, good for growth factor delivery	Poor hydrophilicity, lower bioactivity	Used with growth factors, MSCs, aligned fibers
	PCL	Ductile, bioresorbable	Slow degradation, shape memory	Lower mechanical strength	Used in 3D scaffolds, hybrid structures with gelatin/HA
	Polyurethanes	Highly tunable, good mechanical properties	Easy to modify, bioactivity when seeded	Potential toxicity of by-products	Used with fibroblasts, enhanced by cyclic strain
Hybrid	Natural + Synthetic	Combines bioactivity & strength	Synergistic properties, better scaffold performance	Complexity of fabrication	PLLA/collagen, PCL/gelatin, PLGA/keratin, etc., used for improved tendon regeneration

**Table 2:** Overview of fabrication techniques relevant to tendon tissue engineering.

Technique	Principle	Key Materials	Advantages	Limitations	Relevance to Tendon TE	References
3D printing/Bioprinting	Layer-by-layer deposition of bioinks or biomaterials using computer-aided design	PLA, PCL, GelMA, PRP hydrogels, bioinks with cells	High precision, customizable geometry, tunable porosity, patient-specific scaffolds	Limited vascularization, potential cell damage from pressure/stress	Enables gradient and aligned structures mimicking tendon; supports cell differentiation and mechanical strength	[115,116,117]
Electrospinning	Electric field draws polymer solution into micro/nanofibers; alignment controlled by collector rotation	PCL, gelatin, collagen, GelMA	Produces ECM-like fibrous architecture, tunable alignment, scalable, good cell guidance	Poor pore interconnectivity, limited 3D volume	Aligned fibers guide tenocyte orientation and ECM deposition; coaxial designs combine regeneration + anti-adhesion	[118,119,120]
Freeze-Drying	Freezing polymer solution → sublimation under vacuum → porous 3D scaffold	Collagen, gelatin, silk fibroin, PCL blends	Maintains bioactivity, tunable pore size, simple fabrication, good hydrophilicity	Low mechanical strength, slow production	Creates biocompatible scaffolds and allows gene/drug loading; pore control supports cell infiltration and alignment	[121,122]

## Data Availability

No new data were created or analyzed in this study. Data sharing is not applicable to this article.
